# Sporotrichosis caused by *Sporotrix globosa* in an elderly male farmer at the site of a cat scratch

**DOI:** 10.1016/j.mmcr.2024.100667

**Published:** 2024-09-06

**Authors:** Yuka Sakai, Yuta Norimatsu, Taro Akatsuka, Toshihisa Hamada, Harumi Gomi, Makoto Sugaya

**Affiliations:** a Department of Dermatology, International University of Health and Welfare Narita Hospital, 852 Hatakeda, Narita, Chiba, 286-8520, Japan; b Center for Infectious Diseases, International University of Health and Welfare Narita Hospital, 852 Hatakeda, Narita, Chiba, 286-8520, Japan

**Keywords:** Sporotrichosis, Farmer, Cat scratch scar, *Sporothrix schenckii*, *Sporothrix globosa*

## Abstract

We report a case of sporotrichosis in an elderly male farmer at the site of a cat scratch scar.

An 84-year-old Japanese farmer was scratched by his cat two months before his visit to our hospital.

A skin biopsy was performed. Tissue culture revealed the presence of *Sporothrix globosa*.

The patient was treated with oral itraconazole 200 mg/day for 13 months due to a slow healing ulceration, and the symptoms resolved. (71 words).

## 1 Introduction

The disease sporotrichosis, caused by dimorphic fungi from the *Sporothrix schenckii* complex, is widespread in tropical and subtropical regions [1]. This infection is usually caused by percutaneous trauma in which the fungus invades the host. Such infections may progress to chronic cutaneous, subcutaneous and/or deep infections involving lymphatics, fascia, muscle, cartilage and bone. Sporotrichosis, however, rarely results in death. The pathogen thrives in soil and decaying vegetation such as dead wood, water moss, corn stalks, and hay. Humans are usually infected by traumatic inoculation with the fungus during outdoor activities such as farming, gardening, and animal husbandry. Sporotrichosis is also known as a zoonosis. In particular, cat owners are at risk for developing cat-transmitted sporotrichosis [1]. Recent genetic analysis has shown that there are at least six species of *Sporothrix* [2]. These fungi are known to be distributed geographically, and *Sporothrix globosa* is reported to be the main causative agent in Japan [3].

To the best of our knowledge, there have been no reports of human sporotrichosis transmitted from cats in Japan, although there have been reports of sporotrichosis in cats [4,5].

We report a case of sporotrichosis in the area of a cat scratch scar in an 84-year-old Japanese farmer.

## 2 Case presentation

An 84-year-old Japanese male farmer was scratched by his cat two months before his visit to our hospital. Skin ulcers and nodules had developed and he was treated with gentamicin ointment by the local doctor. As the symptoms did not improve, he was referred to our hospital (day 0). His medical history included hypertension, diabetes mellitus, and benign prostatic hyperplasia.

His medical history included diabetes mellitus and psoriasis vulgaris. He was taking furosemide, febuxostat, doxazosin, nifedipine, empagliflozin, candesartan, vonoprazan, dabigatran etexilate methanesulfonate, and donepezil.

**Fig. 1:**
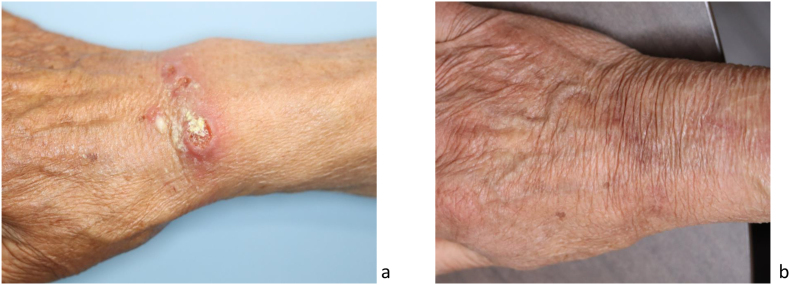
Clinical image of the back of the hand at initial examination and at day 553 (10 months after discontinuation of treatment).

On the dorsum of his right hand were multiple red nodules with ulceration visible (Fig. 1a).

Blood tests at the first visit showed slightly raised C-reactive protein 0.35mg/dL (normal range 0mg/dL-0.3mg/dL), normal white blood cell counts 6180/μL (normal range 3300/μL-8600/μL), and no increased level of β-D glucan (<0.5pg/mL; normal range 0 pg/mL-20 pg/mL). Skin biopsy was performed to differentiate nontuberculous mycobacterial infection and fungal infection (day 0).

**Fig. 2:**
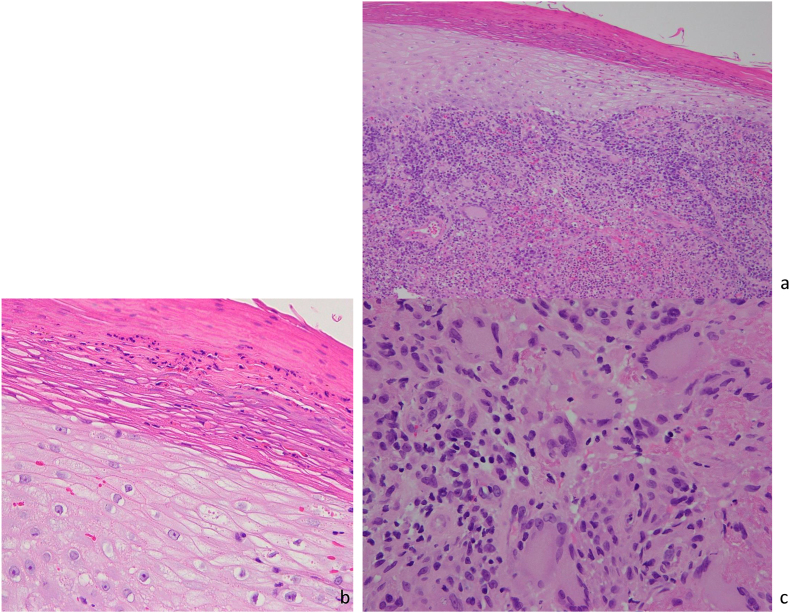
Pathological findings (a) Hematoxin eosin staining showed a diffuse cellular infiltrate spreading into the dermis. (b) Granuloma formation with multinucleated giant cell infiltration was also observed.

Pathologically, hematoxylin and eosin staining showed a diffuse cellular infiltrate spreading into the dermis. Granuloma formation with multinucleated giant cell infiltration was also observed. (Fig. 2a and b).

**Fig. 3:**
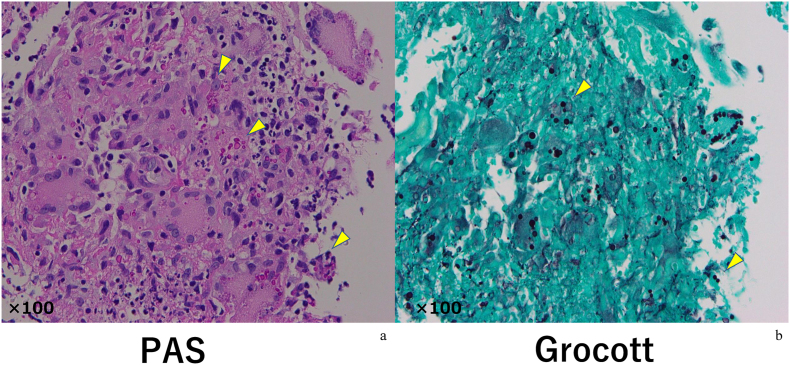
Periodic acid-Schiff stain and Grocott staining Periodic acid-Schiff stain (a) and Grocott staining (b) showed numerous fungi (see arrows).

Grocott staining and Periodic acid-Schiff stain showed numerous fungi (Fig. 3).

**Fig. 4:**
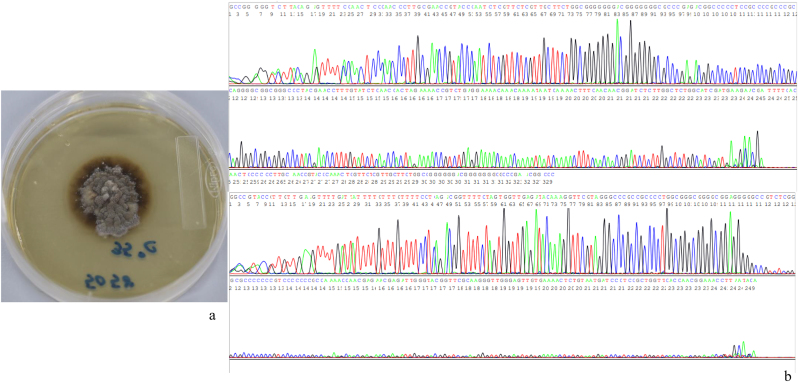
Tissue culture and genetic testing Tissue culture (**Sabouraud agar)** (a) and sequence analysis (amplification of spacer region (ITS1) between 18S rRNA and 5.8S rRNA) (b) led to a diagnosis of infection by *Sporothrix globosa*.

Sequence analysis (amplification of spacer region (ITS1) between 18S rRNA and 5.8S rRNA) led to a diagnosis of infection by *Sporothrix globosa* [6] (Fig. 4).

The patient was treated with itraconazole 200 mg/day for 13 months, e.g. until 4 weeks after the skin laesion was healed and the symptoms resolved. We followed the patient up until day 553, and no recurrences were observed (Fig. 1b).

## 3 Discussion

We present a highly likely cat-transmitted case of sporotrichosis which developed after a cat scratch in an 84-year-old Japanese farmer.

Unfortunately, we were unable to culture any specimen from the cat.

Potassium iodide, itraconazole, and terbinafine are the main drugs used to treat sporotrichosis [1,7,8]. Itraconazole, in particular, is considered the first-line drug for the treatment of sporotrichosis, so it was also used in this case [7]. The patient was treated with itraconazole for a prolonged period for time (13 months) due to a very slow healing ulcer [9,10]. The reason for the slow healing of the skin laesions is most likely related to his poor glycemic control.

*Sporothrix globosa* is also known to be occasionally resistant to itraconazole [[11], [12]], although was not considered in our case as regression of the laesion was observed albeit in a slow manner. No recurrence was noted during the follow-up visit ten months after the discontinuations of the treatment (day 553).

## Conflict of interest

None.

## Ethical form

This study received no funding, and there are no potential conflicts of interest to declare. We obtained written and signed consent to publish the case report from the patient. We have also received permission from the Ethics Committee to report this case. (22-Nr-018).

## CRediT authorship contribution statement

**Yuka Sakai:** Writing – review & editing, Writing – original draft, Visualization, Resources, Investigation, Data curation. **Yuta Norimatsu:** Writing – review & editing, Writing – original draft, Visualization, Validation, Supervision, Software, Resources, Project administration, Methodology, Investigation, Formal analysis, Data curation, Conceptualization. **Taro Akatsuka:** Writing – review & editing, Visualization, Resources, Data curation. **Toshihisa Hamada:** Writing – review & editing, Visualization, Resources, Methodology, Data curation. **Harumi Gomi:** Writing – review & editing, Validation, Supervision, Resources, Methodology, Investigation, Formal analysis, Conceptualization. **Makoto Sugaya:** Writing – review & editing, Supervision, Software, Resources, Project administration, Methodology, Conceptualization.
