# Supplementary figures and images for: Sporotrichosis caused by Sporotrix globosa in an elderly male farmer at the site of a cat scratch
Source: Med Mycol Case Rep. 2024 Sep 6;46:100667. doi: 10.1016/j.mmcr.2024.100667 (PMC11416248; doi:10.1016/j.mmcr.2024.100667)

## Slide 1
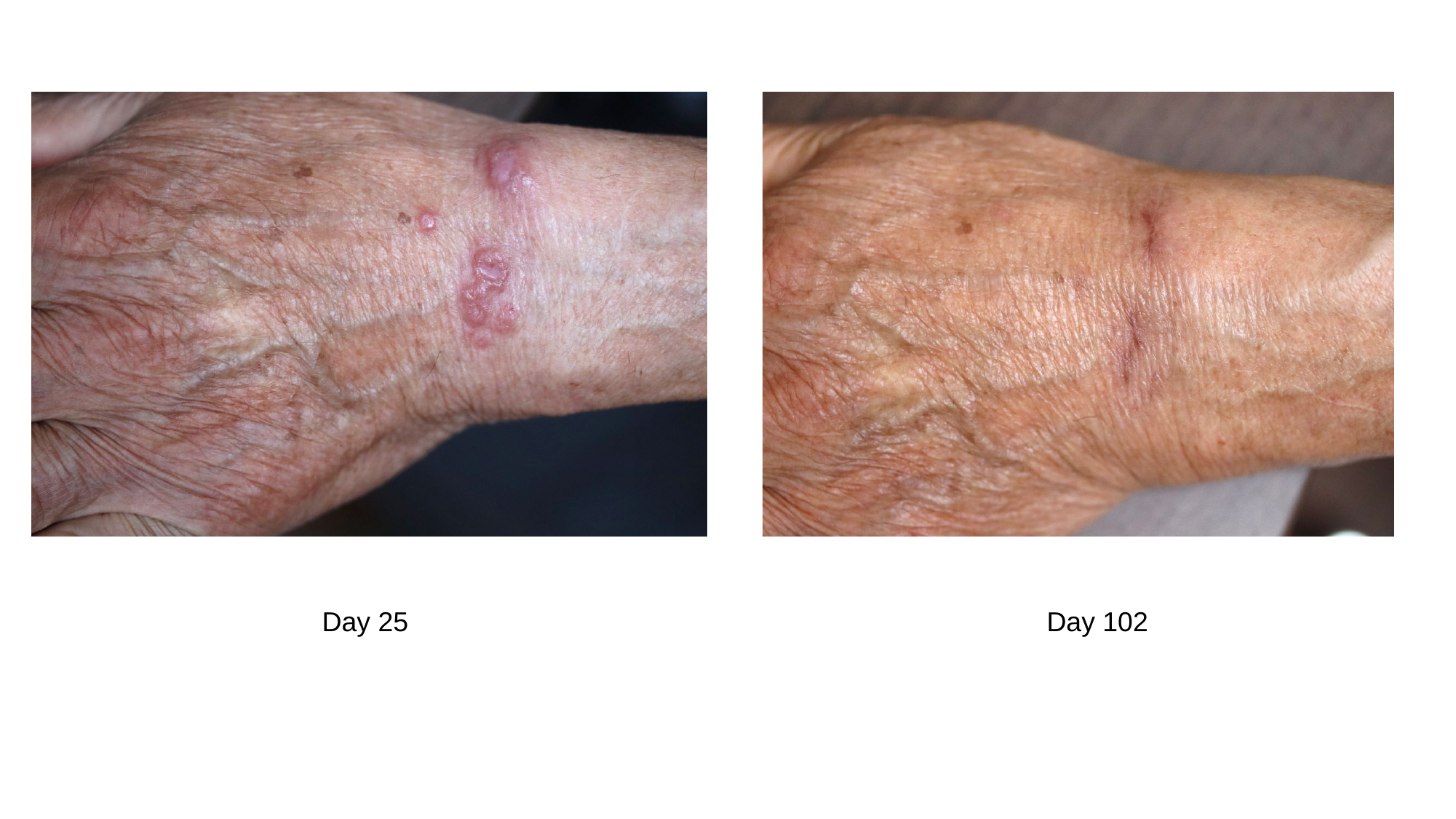

Day 25
Day 102

Supplement: Multimedia component 1 [file mmc1.pptx]
